# Diarrheal Illness and Healthcare Seeking Behavior among a Population at High Risk for Diarrhea in Dhaka, Bangladesh

**DOI:** 10.1371/journal.pone.0130105

**Published:** 2015-06-29

**Authors:** Fahima Chowdhury, Iqbal Ansary Khan, Sweta Patel, Ashraf Uddin Siddiq, Nirod Chandra Saha, Ashraful I. Khan, Amit Saha, Alejandro Cravioto, John Clemens, Firdausi Qadri, Mohammad Ali

**Affiliations:** 1 International Centre for Diarrheal Disease Research, Dhaka, Bangladesh; 2 International Vaccine Institute, Seoul, Korea; 3 Johns Hopkins Bloomberg School of Public Health, Baltimore, Maryland, USA; 4 UCLA Fielding School of Public Health, Los Angeles, CA, United States of America; Hunter College, UNITED STATES

## Abstract

Diarrhea remains one of the major causes of death in Bangladesh. We studied diarrheal disease risk and healthcare seeking behavior among populations at high risk for diarrhea in Dhaka, Bangladesh. Data were obtained from a cross-sectional survey conducted during April and September 2010. The prevalence of diarrhea was calculated by age-group and sex. A generalized estimating equation with logit link function was used to predict diarrheal disease risk and seeking care from a professional healthcare provider. Of 316,766 individuals, 10% were young children (<5 years). The prevalence of diarrhea was 16 per 1000 persons among all ages; young children accounted for 44 per 1000 persons. Prevalence of diarrhea was significantly higher (p=.003) among younger males (<15 years) compared to that among younger females. In contrast, prevalence of diarrhea was significantly higher (p<.0001) among older females (≥15 years) compared to that among older males. An increased risk for diarrhea was observed in young children, males, and those staying in rented houses, lower family members in the house, using non-sanitary toilets, living in the area for short times, living in a community with less educated persons, living in a community with less use of safe water source for drinking, or living close to the hospital. About 80% of those with diarrhea sought care initially from a non-professional healthcare provider. Choice of the professional healthcare provider was driven by age of the patient, educational status of the household head, and hygienic practices by the household. The study reaffirms that young children are at greater risk for diarrhea. Like other developing countries most people in this impoverished setting of Dhaka are less likely to seek care from a professional healthcare provider than from a non-professional healthcare provider, which could be attributed to a higher number of diarrheal deaths among young children in Bangladesh. Dissemination of information on health education, increasing the supply of skilled healthcare providers, and low-cost and quality healthcare services may encourage more people to seek care from professional healthcare providers, thus may help reduce child mortality in the country. Further studies are warranted to validate the results.

## Background

Globally diarrheal disease is the second leading cause of death in children under five, and responsible for the deaths of about 760,000 children every year [1]. The disease is particularly dangerous for young children, who are more susceptible to dehydration and nutritional losses, especially those living in low and middle income countries. The true burden of diarrheal illness among populations at high risk in Bangladesh has not been well evaluated, and only a few studies [2, 3] were conducted to examine the healthcare seeking behavior for diarrhea, which has important implications on the outcomes of diarrheal diseases.

Healthcare seeking behavior is any activity undertaken by individuals for the purpose of finding an appropriate treatment in the event of illness [4, 5]. The behavior depends on several factors that include historical patterns of services used, illness type and severity, pre-existing beliefs about illness causation, accessibility of service options, convenience, and quality of service provision, as well as age, gender, and social status of the sick persons [6–11]. Usually, an individual from an impoverished setting seeks informal healthcare services to save time and money. However, there is inadequate knowledge of what keeps individuals away from professional healthcare providers [12]. Identification of the factors that may facilitate or impede the use of appropriate healthcare services may help identify those who are most vulnerable, and provide information that policy makers can use to target their services to those in greatest need.

In developing countries, diarrhea is often inadequately managed at home [13], which may result in poor outcomes. Timely medical attention for diarrhea is important for reducing deaths from diarrhea [14]. Understanding the disease risk and identifying determinants of seeking care from a professional healthcare provider may provide a basis upon which governments can estimate the burden of the disease and detect barriers to seeking appropriate healthcare. This is particularly important in countries like Bangladesh, where a large proportion of the population is below the poverty line. This study has been designed to estimate the burden of diarrhea illness, to identify risk factors for diarrhea, and predictors for seeking care from professional healthcare providers in a high risk population in Dhaka, Bangladesh.

## Methods

### The study area

**Fig 1:**
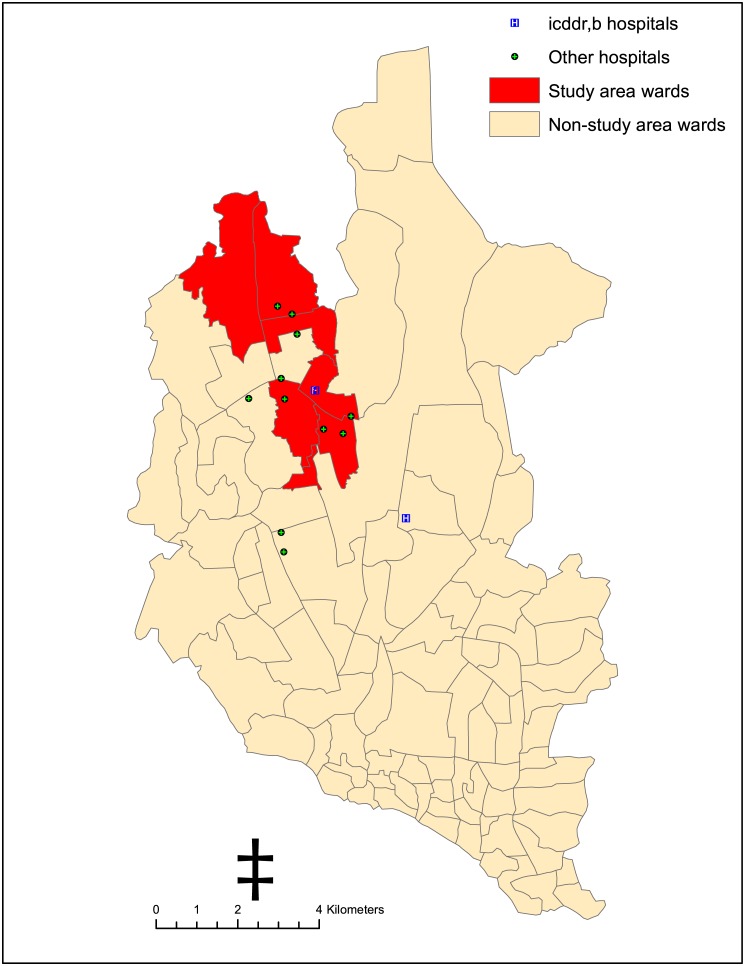
The study area and the catchment area hospitals in Dhaka, Bangladesh.

To prevent the high risk population from risk for cholera in Bangladesh, a cholera vaccination demonstration project, “Introduction of Cholera Vaccine in Bangladesh (ICVB),” was launched in close collaboration with government authorities to provide a new low-cost cholera vaccine through mechanisms similar to what the government of Bangladesh uses during its mass polio and measles vaccine campaigns. The project was conducted in six wards of Mirpur, Dhaka, Bangladesh (Fig 1) targeting people at high risk for diarrhea, which was defined using five criteria: overcrowding, poor sanitation, unhealthy and unhygienic living condition, unsafe water use, and low income dwelling.

Overcrowding was defined as three or more adults living in one room. A poor sanitation is defined as the lack of a sanitary latrine such as pour flash latrine, water sealed latrine, or improved pit latrine as well as with direct connection to a sewer line or septic tank. Shared water sources, kitchens, or toilets were defined as unhealthy and unhygienic living conditions. Unsafe water use was defined as the lack of clean water for drinking and for washing utensils. Low income dwellings were defined as such if they were constructed with poor or non-permanent construction materials like bamboo, cheap wood, or scraps. The overall condition of each dwelling was assessed by field interviewers; those dwellings with one or more of the above characteristics were categorized as high risk households and included in the population enumeration. To validate the accuracy of correct identification of the high risk population, we conducted a sample survey in which we targeted 100 households using simple random sampling from each ward (a total of 600 households in the six wards). The survey was carried out one month after the census was over by different groups of field workers. The survey was ended up with 579 households from the 6 wards, and the outcomes of the survey suggested 95% of the interviewers accurately identified the target households.

### Health systems in Bangladesh

Bangladesh has a health system that is run by public and private sectors. The private sector is run by local entrepreneurs, a variety of NGOs and international organizations. The public sector is largely used for out-patient, in-patient and preventive care, while the private sector is used largely for outpatient and in-patient curative care. Over the last decade, Bangladesh has made significant improvement in health sector, which make it an example for other developing countries even though being a resource poor country [15]. Despite this, health systems in Bangladesh are ineffective in providing care for all the citizens resulting in inequalities in health and health care utilization between different groups. There is also a shortage of over 60,000 doctors and 140,000 nurses [16]. The nurse-physician ratio is also very low—only one nurse per three physicians, while the ratio should other way around, as recommended by WHO. Absenteeism of physicians and lack of drugs, supplies and other facilities are also common in the health complexes. As a result, the publicly funded healthcare system is used by only 25% of the population. Furthermore, Bangladesh has an acute shortage of medical technologists and allied health professionals such as physiotherapists, laboratory assistants, and x-ray technicians, among others. Thus, a majority of the country’s population (up to 80%) seek their first line of care from informal healthcare providers: traditional healers, faith healers and community health workers(16).

### The study data

From April to September 2010, a *de jure* census was carried out by the project staff to enumerate regular residents of the area. The census survey was conducted using a paperless system [17]; the data were entered directly into personal digital assistants (PDAs) during interviews. Verbal informed consent was obtained prior to the interviews.

The geographic locations of the households as well as other geographic features of interest including health clinics were also collected during the census survey, and from this we created a geographic information system (GIS) database. A unique ID was assigned to each member of the enumerated households. The respondent was the head of household or an adult member of household who was able to give all information of all household members. Relevant demographic and socioeconomic information, as well as information on diarrhea prevalence in the past 48 hours, were collected during the census survey. Consistent with WHO guidelines [18], diarrhea was defined as 3 or more loose stools in 24 hours.

Surveyed subjects reporting diarrhea were also asked about utilization of healthcare practitioners and facilities. Since healthcare for an episode may be sought multiple times, we kept provision for collecting this information for a maximum of three visits, and accordingly the choice of healthcare in each visit was collected. Information on subsequent visits was asked when the patient was not cured after a visit was made.

### Variable definitions

Healthcare providers were classified into two categories: professional and non-professional. The professional category encompassed MBBS certified doctors and staff from icddr,b hospital, Mirpur Treatment Centre, or other clinics or hospitals. Non-professional providers included home treatment, pharmacies, ayurvedic healers, homeopathic healers, quacks (defined as those practicing allopathic medicine without a professional degree), or any other healthcare providers without medical degrees.

Less than five years of schooling was defined as less education. Safe drinking water source was defined as one’s own tap or bottled mineral water. Boiling, filtering, or adding chemicals in water were considered as treated water. GIS mapping of the households was used to obtain population density, and the rates of educated people (individuals ≥12 years were used in this population considering minimum five years of schooling is required for being educated according to our definition) and proportion of people using a safe water source around 100m of the household. The GIS data of the households allowed us to compute distance from household to the icddr,b hospital. The 100m radius was arbitrarily chosen considering that the particular radius would be appropriate in a highly densely population setting like our study area.

### Data analysis

We assessed disease risk and the choice of healthcare services in the event of diarrhea using generalized estimating equations (GEE) with the logit link function and independent and exchangeable within-household correlation matrixes [19], considering that the data were correlated at the household level. Initially, we fitted bivariate models with each one of the variables. The GEE took the occurrence of reported diarrhea as well as healthcare utilization in each analyzed individual as the dependent variable and the variables that showed significant association (P<0.10) with each of the outcomes in the bivariate model were selected for the final model. Exponents of the coefficients of independent variables in the models were used to estimate the odds ratio (OR) of reported diarrhea or the choice of healthcare. Standard errors for the coefficients were used to estimate p-values and associated 95% confidence intervals (95% CI) for the ORs. In a bivariate model, we examined association of each variable with the outcome, and selected the variables that showed significant at p<0.10 for the final model. The data file along with the data dictionary that was used in this study has been provided as supporting documents (see S1 and S2 Dataset). All statistical tests were interpreted in a two-tailed fashion. Analyses were performed using SAS Version 9.3.

### Role of the funding source

The project was funded by the Bill & Melinda Gates Foundation. They provided inputs in the design and planning of the study, but had no role in data collection, data analysis, data interpretation, or writing of the report. The corresponding author had full access to all the data in the study and had final responsibility for the decision to submit the manuscript for publication.

## Ethics

The study was approved by the Research Review Committee and Ethical Review Committee of icddr,b as well as by the Institutional Review Board (IRB) of the International Vaccine Institute (IVI). According to the icddr,b and IVI IRBs, we required verbal consent only from the households who participated in the baseline census survey due to the large sample size and lack of literacy among most of the participants. The census staff reported verbal consent of the participants or the adult respondent of each household by ticking the appropriate spot in the census questionnaire.

## Results

A total of 316,766 individuals in 79,438 households were enumerated in the census survey. Among these individuals, 32,692 (10%) were under five years of age. About 48% of this population was female. The median family size was 4, and the median amount of time that the surveyed households have been living in their current residence was 6 years. In total 5,156 individuals reported to have diarrhea in last 48 hours prior to interview yielding a prevalence of 16 per 1000 persons (Table 1). Among individuals who reported having diarrhea, 28% of them were young children (<5 years old). The prevalence of diarrhea in this age-group was 44 per 1000 persons; whereas it was 14 per 1000 persons among individuals 5 to 14 years of age. Among individuals 15 years and older, the rate was 13 per 1000 persons. The results also show that the rate was significantly higher among males under 15 years (25.7/1000) compared to that among females (22.8/1000) in the same age-group (p = .003). In contrast, the rate was significantly higher among females 15 years and older (14.2/1000) compared to that among males (11.3/1000) in that age-group (p < .0001).

**Table 1:** Prevalence of diarrhea by age and sex groups among a high risk population in Dhaka, Bangladesh.

ge groups	Male			Female			All sexes		
	# persons	# diarrhea cases	Rate/1000	# persons	# diarrhea cases	Rate/1000	# persons	# diarrhea cases	Rate/1000
<5 years	16,399	761	46.4	16,292	683	41.9	32,691	1,444	44.2
5-<15 years	31,433	469	14.9	31,619	408	12.9	63,052	877	13.9
<15 years	47,832	1230	25.7	47,911	1091	22.8	95,743	2,321	24.24
15 years +	106,111	1,203	11.3	114,912	1,632	14.2	221,023	2,835	12.8
All ages	153,943	2,433	15.8	162,823	2,723	10.0	316,766	5,156	16.3

The chi-square test for evaluating the difference in the rates between male and female yielded p = .049 for subjects <5 years, p = .031 for subjects 5-<15 years, p = .003 for subjects <15 years, and p < .0001 for subjects 15 years and older. Overall, the difference in the rates between male and female was significant at p = .041.

Almost all demographic and socioeconomic characteristics varied significantly between individuals reporting diarrhea and presumably non-diarrhea (Table 2). The results for identifying risk factors for diarrhea using generalized estimating equation (GEE) after accounting for within household-level correlation are presented in Table 3. An increased risk for diarrhea was observed among young children, males, those staying in a rented house, lower family members in the house, those using a non-sanitary toilet, those living in a community (within 100m around the household) with a less educated adult or less use of their own water source for drinking, and those living in shorter distance to the hospital.

**Table 2:** Comparison of individual and household level characteristics between those who 'reported diarrhea' and those who presumably did not have diarrhea.

Variables	Reported diarrhea (n = 5,156)	Presumably did not have diarrhea (n = 311,610)	p-value*
**Binary variables**			
Age less than 5 years	1,444 (28.0%)	31,247 (10.0%)	< .0001
Female	2,433 (47.2%)	151,510 (48.6%)	0.0335
No or less educated household head	2,728 (52.9%)	171,203 (54.9%)	0.0093
Living in not owned house	4,453 (86.4%)	248,137 (79.6%)	< .0001
Four or more persons in the household	1,214 (23.6%)	86,792 (27.8%)	< .0001
Single room	4,491 (87.1%)	254,925 (81.8%)	< .0001
Non-sanitary toilet	1,248 (24.2%)	70,545 (22.6%)	0.0161
Safe water source for drinking	4,888 (94.8%)	292,234 (93.8%)	0.0134
Treating drinking water	2,327 (45.1%)	145,377 (46.6%)	0.0688
Non-availability of soap for hand washing in the house (based on observation)	466 (9.0%)	25,286 (8.1%)	0.0401
**Continuous variables**			
Age of the household head (years)	38.21 (11.98)	40.1 2 (12.29)	< .0001
Living in the area (years)	4.35 (8.28)	5.96 (9.67)	< .0001
Population density within 100m of the household	1818.07 (897.53)	1876.33 (933.60)	< .0001
Percent of educated people within 100m of the household	0.51 (0.11)	0.50 (0.12)	0.0255
Percent people around 100m of household used own water source for drinking	0.06 (0.05)	0.06 (0.05)	0.0048
Distance from household to ICDDR,B hospital (km)	1.63 (0.82)	1.81 (0.83)	< .0001

Note, number and percentage (in parenthesis) are shown or the binary variables and mean and standard deviation (in parenthesis) are shown for the continuous variables. Missing values were addressed in these calculations.

*The p-values were derived using generalized estimating equation after adjusted for household level clustering.

**Table 3:** Predictors of the diarrheal illness among a high risk population in Dhaka, Bangladesh, 2010.

Variables	Odds ratio	95% CI	p-value
Age less than 5 years	3.46	3.25–3.68	< .0001
Female	0.93	0.88–0.99	0.0140
Living in not owned house	1.20	1.07–1.33	0.0012
Four or more persons in the household	0.88	0.81–0.96	0.0030
Single room	1.10	0.99–1.22	0.0569
Non-sanitary toilet	1.11	1.03–1.20	0.0063
Living in the area (years)	0.99	0.98–0.99	< .0001
Percent of educated people within 100m of household	0.74	0.54–0.99	0.0462
Percent people around 100m of household used own water source for drinking	0.40	0.21–0.75	0.0041
Distance from household to ICDDR,B hospital (km)	0.75	0.72–0.781	< .0001

* Odds ratio for the cited variable, adjusted for household level clustering as well as all other variables in the table, calculated in a model using Generalized Estimating Equations (GEE) with the logit link function.

**Fig 2:**
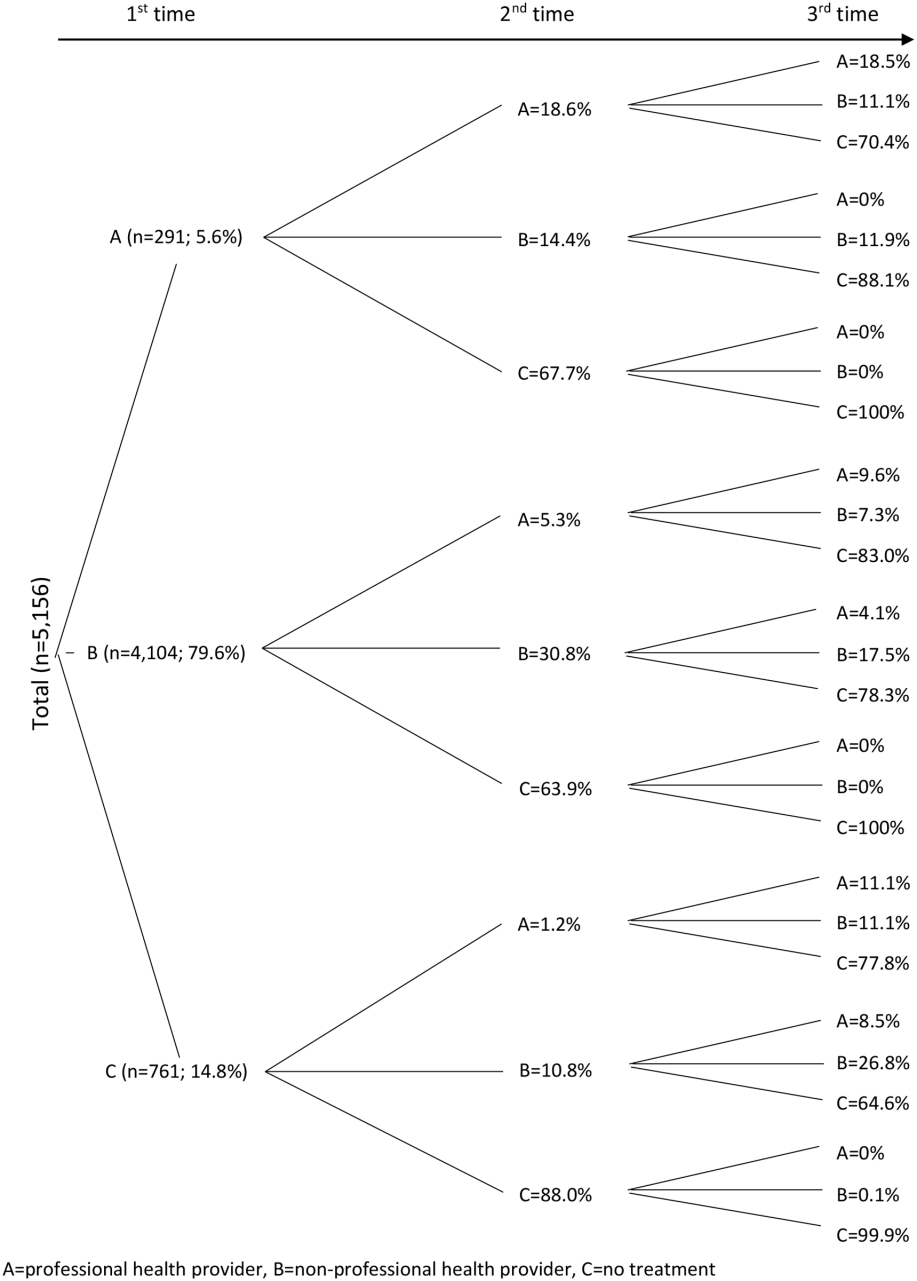
Healthcare utilization patterns of subjects reporting diarrhea in 48 hours prior to interview, Mirpur, Dhaka, Bangladesh, 2010.

Of the 5,156 subjects reporting diarrhea, 6% first sought care from a professional healthcare provider (A), 80% from a non-professional healthcare provider (B), and 15% did not seek treatment at all (C) (Fig 2). The majority of subjects in each category did not seek healthcare (68%, 64%, and 88%, for A, B, and C, respectively) a second time. Of those who sought healthcare initially from a non-professional healthcare provider, 31% returned to a non-professional and only 5% went to a professional healthcare provider. Of the people who initially sought care from a professional healthcare provider, 19% visited a professional and 14% a non-professional healthcare provider for the second time. Of the 15% of subjects who initially sought no treatment, 11% sought care from a non-professional and only 1% from a professional healthcare provider. The trend of seeking healthcare from the non-professional healthcare providers remained the same for the third time seeking care (Fig 2).

The characteristics of the persons who initially sought care from professional and non-professional healthcare providers are shown in Table 4. The results of the multivariable model after adjusting for household-level correlation yielded young children, persons living in a household having an educated household head, persons living in a household not using treated water for drinking, and persons living in a household where soap was present (based on observation) for hand washing had sought their care from a professional healthcare provider (Table 5).

**Table 4:** Comparison of individual and household characteristics among people sought their medical care for diarrhea from professional and non-professional healthcare providers, Dhaka, Bangladesh, 2010.

Variables	Professional health provider (n = 291)	Non-professional health provider (n = 4,109)	p-value*
**Binary variables**			
Age less than 5 years	179 (61.5%)	1,063 (25.9%)	< .0001
Female	133 (45.7%)	1,989 (48.5%)	0.4997
No or less educated household head	109 (37.5%)	2,173 (52.9%)	< .0001
Living in not owned house	252 (86.6%)	3,545 (86.4%)	0.9165
Four or more persons in the household	69 (23.7%)	962 (23.4%)	0.7881
Single room	245 (84.2%)	3,579 (87.2%)	0.1279
Non-sanitary toilet	63 (21.6%)	988 (24.1%)	0.4606
Safe water source for drinking	274 (94.2%)	3,889 (94.8%)	0.6715
Treating drinking water	86 (29.5%)	1,815 (44.2%)	< .0001
Non-availability of soap for hand washing in the house (based on observation)	37 (12.7%)	369 (9%)	0.0318
**Continuous variables**			
Age of the household head (years)	35.76 (11.42)	38.40 (11.93)	0.0008
Living in the area (years)	4.15 (7.88)	4.40 (8.41)	0.6329
Population density within 100m of the household	1677.36 (846.13)	1790.64 (893.25)	0.0425
Percent of educated people within 100m of the household	0.53 (0.11)	0.51 (0.11)	0.0103
Percent people around 100m of household used own water source for drinking	0.06 (0.04)	0.06 (0.05)	0.5887
Distance from household to ICDDR,B hospital (km)	1.52 (0.77)	1.60 (0.82)	0.0870

Note, number and percentage (in parenthesis) are shown or the binary variables and mean and standard deviation (in parenthesis) were shown for the continuous variables. Missing values were addressed in these calculations.

*The p-values were derived using generalized estimating equation after adjusted for household level clustering.

**Table 5:** Predictors of seeking care from professional healthcare providers among high risk population for diarrhea in Dhaka, Bangladesh.

Variables	Odds ratio	95% CI	p-value
Age less than 5 years	4.15	3.24–5.29	< .0001
No or less educated household head	0.59	0.45–0.77	0.0001
Treating drinking water	0.65	0.49–0.85	0.0020
Availability of soap for hand washing in the house	1.54	1.05–2.25	0.0258

* Odds ratio for the cited variable, adjusted for household level clustering as well as all other variables in the table, calculated in a model using Generalized Estimating Equations (GEE) with the logit link function.

## Discussion

We examined the burden, risk factors for diarrhea, and patterns of healthcare utilization in a high risk population in Dhaka, Bangladesh. The prevalence of diarrhea in this setting was very high (16 per thousand persons). However, we did conduct the survey during April to September when the diarrheal disease burden is generally at its higher levels in the year [20, 21]. Our results also show that the risk for diarrhea is higher for young children (<5 years) than that for older individuals. This is not unexpected, as diarrheal diseases comprise the second leading cause of death and morbidity worldwide in young children [22]. This increased risk for diarrhea is likely the product of multiple issues such as immune naivety, breastfeeding practices, malnutrition and a multitude of other factors. Although the prevalence of diarrhea was higher among young children, the burden was higher among older individuals, as they account for the majority of the population. The observation that adult women are at a greater risk than adult men could be due to their having greater access to unsafe water for the household activities. The reason behind higher prevalence among young males compared to that among young females could be due to young males are more likely to wander off in unsanitary surroundings compared to young females. One study conducted at icddr,b hospital shows that males are admitted at a higher proportion than females to the ICU (64% vs 36%) with diarrhea [23]. Another study in Bangladesh shows where free healthcare is available, equal number of males and females who were affected with diarrhea, 66 percent more males than females *were* taken to health facilities for treatment [24]. These findings are similar to another study conducted in Sudan where males are 3% more likely to have diarrhea compared to females under the age of five years [25].

We detected an increased risk for diarrhea among those who lived in a rented house as opposed to an owned house, and those using a non-sanitary toilet. A study on shigellosis in Thailand found people living in rented homes were at a much higher risk for shigellosis than those living in homes they owned [26]. Home ownership status used as a proxy for socioeconomic status and wealth has been positively correlated with health status [27, 28]. Thus, it is possible that those living in rented dwellings were more impoverished than those who owned their dwellings, which in turn predisposed them to diarrheal illness. Those who were living in the area for a longer period of time were less likely to have the disease compared to their shorter term counterparts. They may have usual source of health care facility and have good relationship with the local doctors due to their longer stay in the area. Understanding the impact of the socioecological context on diarrheal illness is important, because it is not only the individual’s own status, but the community characteristics that may also influence the risk of getting a disease. In our study, we observed those who lived in a community where higher numbers of educated people were residing or where higher numbers of people used safe water for drinking were less likely to have diarrhea. This indicates health knowledge and health behavior of the community are important for reducing transmission of the disease in the community. As observed in other studies [29, 30], we also found higher levels of reporting of diarrheal illness among people living proximate to the hospitals in our study.

In our study, we observed that a majority of the participants (80%) sought their care first from a non-professional healthcare provider. Even after failing to recover from the treatment offered by the first healthcare provider, they were less likely to visit to a professional healthcare provider as the second or third choice. The reason for not seeking professional healthcare could be the perception that the episodes were not serious enough [2]. The other cause could be due to poor people in Bangladesh being especially disadvantaged in accessing quality healthcare because of their marginalized position in society. Evidence also shows that there is pro-rich bias in the distribution of health benefits, even for simple and cheap health interventions [31–33]. Only 6% of the patients visited to a professional healthcare provider probably due to having moderate or severe diarrhea. Those who sought care first from a professional or non-professional healthcare provider, about 65% of them did not seek further care from a provider suggesting end of the episode. Those who did not seek any treatment at the beginning of episode, 88% of them did not seek care from a healthcare provider as the 2^nd^ or 3^rd^ choice suggesting the episodes were mild and was probably cured after self-treatment such as intake of oral rehydration solution (ORS), which is commonly used at home in Bangladesh in the event of diarrhea. Only a few people had to seek healthcare for the third time, meaning they had longer duration of the episode. The results of the analysis illustrate that the healthcare-seeking behavior could be treated as a proxy for diarrhea severity [34] as well as outcome pattern of the illness.

We also found that children with diarrheal illness were more likely to be brought to a professional healthcare provider than older individuals. The greater investment in time and money to treat children is perhaps best explained by the perceived higher vulnerability of children compared to older individuals. It is surprising that those who used treated water for drinking were less likely to visit to a professional healthcare provider than those who used untreated water. Perhaps by using treated water, these individuals thought that their illness was not serious enough that they needed to go to a professional healthcare provider. On the other hand, those who used soap for hand-washing were more likely to visit to a professional healthcare provider probably due to their health consciousness. Not owning home where the household is living in, a proxy for poverty, did not appear to play a significant role in the healthcare choices among the high risk population for diarrhea in the study area where only 14% of the households owned the house. If we would have the data on household income where there could be a greater variability, and that might yield to a different result. However, we did not collect the data on household income, because this is a sensitive question to the people in that community, and the response would not be reliable. Education of the household head played a major role for the choice of professional health care provider, probably due to their understanding of potential benefit from professional healthcare providers in the event of illness [35]. These findings divulges that age, gender, educational level, living condition, life style, socioeconomic status, distance to health care clinic are some barriers to accessing professional healthcare in our study.

Our study has several limitations. Usually, people seek healthcare service in the event of severe diarrhea [2]. The limitation in this study is that we did not collect the information on severity of the episode, because it was reported diarrhea and the assessment of severity by the respondents would not be reliable as observed in a study conducted in Bangladesh [2]. In addition, the definition of diarrhea in this study was based on subject’s history instead of a clinical or microbiological diagnosis. Consequently, the term “diarrhea” could have encompassed a wide spectrum of illness. It is also possible that milder cases were not reported. Additionally, only one adult respondent provided the diarrheal information of all the members in the household, and the respondent might have been unaware of or unwilling to discuss the gastrointestinal complaints of their household members. This could be one of the reasons that the household size is negatively associated with the outcome. We also note that the measures of health-seeking behavior were based on reported illness and treatment action, and not directly observed as the illness process unfolded. However, by limiting the recall period to the past 48 hours, attempts were made to minimize problems of inaccurate recall arising from retrospective records [36, 37]. Another limitation relates to the possible effect of illness stage on treatment choice; that more advanced illness may be treated differently than early stage disease where home and folk remedies may initially suffice [38]. Fortunately, the cross-sectional nature of the study, and the inclusion of all reported illness occurring in the last 48 hours irrespective of severity, helps obviate the potential confounding influence of illness stage in analysis.

## Conclusion

With 14 million inhabitants, Dhaka is one of the world’s megacities, and many of its residents lack access to safe water and sanitation. We found a high burden of diarrheal illness in the impoverished part of the population. Several factors were associated with the risk for diarrhea, and we observed a significant proportion of people with diarrheal illness continue to seek care from unqualified healthcare practitioners. The challenges faced in Dhaka are common to congested urban areas worldwide, as are the trends seen in diarrheal risk and healthcare utilization. The significant deaths due to diarrheal illness, especially in children under five, highlights the need for seeking care from professional healthcare providers. How that can be encouraged remains an important challenge. Previous research indicates that investing in expanded health care service delivery does not necessarily increase use of services [39–41]. Increasing access to professional healthcare should take on both expanding delivery of the healthcare services and addressing the factors influencing individuals’ health care seeking behavior. We have identified some barriers to accessing professional healthcare in our study. Dissemination of information on health education, increasing the supply of skilled healthcare providers, and low-cost and quality healthcare services may encourage more people to seek care from professional healthcare providers, thus may help reduce child mortality in the country. Further studies are warranted to validate the results.

## Supporting Information

S1 Dataset The data file used in the analysis. (ZIP)

S2 Dataset The data dictionary of the file used in the analysis. (PDF)
