# Supplementary material for: Diarrheal Illness and Healthcare Seeking Behavior among a Population at High Risk for Diarrhea in Dhaka, Bangladesh
Source: PLoS One. 2015 Jun 29;10(6):e0130105. doi: 10.1371/journal.pone.0130105 (PMC4485467; doi:10.1371/journal.pone.0130105)
Supplement: S2 Dataset — (PDF) [file pone.0130105.s002.pdf]

# Data Dictionary

| Table | Variable name | Description of variable | Data type | Width | Coding category of variable |
|---|---|---|---|---|---|
| Datafile | HH | Household ID (1-2 digits : Cluster 3-4 digits : Block 5-8 digits : serial of households within cluster and block) | Character | 8 | Exact value |
| Datafile | Slno | Member serial number | Character | 2 | Exact value |
| Datafile | RTH | Relation to household head | Numeric | 2 | 1-Household head |
| | | | | | 2-Spouse of head |
| | | | | | 3-Son/daughter |
| | | | | | 4-Son/daughter-in-law |
| | | | | | 5-Brother/sister |
| | | | | | 6-Brother/sister-in-law |
| | | | | | 7-Father/mother |
| | | | | | 8-Father/mother-in-law |
| | | | | | 9-Grand-son/grand-daughter |
| | | | | | 10-Grand/father/mother |
| | | | | | 11-Other relation |
| | | | | | 12-Servent |
| | | | | | 13-No relation |
| Datafile | Sex | Sex | Numeric | 1 | 1-Male |
| | | | | | 2-Female |
| Datafile | Bdate | Date of birth | Date | 8 | Exact value |
| Datafile | Age | Age in years | Numeric | 3 | Exact value |
| Datafile | Edu | Education | Numeric | 2 | 1-Class 1 passed |
| | | | | | 2-Class 2 passed |
| | | | | | 3-Class 3 passed |
| | | | | | 4-Class 4 passed |
| | | | | | 5-Class 5 passed |
| | | | | | 6-Class 6 passed |
| | | | | | 7-Class 7 passed |
| | | | | | 8-Class 8 passed |
| | | | | | 9-Class 9 passed |
| | | | | | 10-SSC passed |
| | | | | | 12-HSC passed |
| | | | | | 14-BA/BCOM/BSC passed |
| | | | | | 16-Hons/MBBS/BSC Eng passed |

| Table | Variable name | Description of variable | Data type | Width | Coding category of variable |
|---|---|---|---|---|---|
| | | | | | 17-MA/MSC/MCOM/MS/MD/FCPS passed<br>77-No formal education,<br>88-No education |
| Datafile | Ocp | Occupation | Numeric | 2 | 1-Unemployed<br>2-Housewife<br>3-Beggar<br>4-Pensioners<br>5-Household helping hand<br>6-Driver<br>7-Rickshaw/van/cart puller<br>8-Daily wage earner/laborer<br>9-Farmer/fisherman<br>10-Tailor/barber/craftsman<br>11-Traders/business owner<br>12-Service<br>13-Teacher<br>14-Doctor<br>15-Engineer<br>16-Paid/unpaid apprentice<br>17-Student<br>18-Hawker<br>77-Others<br>99-Unknown |
| Datafile | Dia48 | Diarrhea within 48 hours | Numeric | 1 | 1-Yes<br>2-No |
| Datafile | HCare1 | Health care utilization: 1 | Numeric | 2 | 1-Home treatment<br>2-MBBS doctor<br>3-ICDDR,B hospital<br>4-Mirpur Treatment Centre<br>5-Other clinic/hospital<br>6-Pharmacy<br>7-Homeopathy<br>8-Ayurbedic<br>9-Quack<br>77-Other<br>99-No treatment |
| Datafile | Hcare2 | Health care utilization: 2 | Numeric | 2 | Same as Hcare1 |

| Table | Variable name | Description of variable | Data type | Width | Coding category of variable |
|---|---|---|---|---|---|
| Datafile | Hcare3 | Health care utilization: 3 | Numeric | 2 | Same as Hcare1 |
| Datafile | Hhage | Household head age in years | Numeric | 3 | Exact value |
| Datafile | Hhedu | Household head education | Numeric | 2 | Same as Edu |
| Datafile | Hhsex | Household Head sex | Numeric | 1 | 1-Male |
| | | | | | 2-Female |
| Datafile | HhType | Type of household ownership | Numeric | 1 | 1- Own |
| | | | | | 2-Rented |
| | | | | | 3-Supplied by employer |
| Datafile | Hhsize | Household members | Numeric | 2 | Exact Value |
| Datafile | LiveMo | How many months have you been living here? | Numeric | 2 | Exact value |
| Datafile | Rooms | Number of rooms | Numeric | 2 | Exact value |
| Datafile | TAdult | Adult toilet type | Numeric | 1 | 1-Sanitary with flush |
| | | | | | 2-Sanitary without flush |
| | | | | | 3-Non-sanitary |
| | | | | | 4-Use open space |
| Datafile | TChild | Child toilet type | Numeric | 1 | 1-Sanitary with flush |
| | | | | | 2-Sanitary without flush |
| | | | | | 3-Non-sanitary |
| | | | | | 4-Use open space |
| | | | | | 5-Use plastic/cane pot |
| | | | | | 8-No <5 years child in the HH |
| Datafile | DrinWa | Source of drinking water | Numeric | 2 | 1-Own tap |
| | | | | | 2-Own well |
| | | | | | 3-Own hand pump |
| | | | | | 4-Communal tap |
| | | | | | 5-Communal well |
| | | | | | 6-Communal hand pump |
| | | | | | 7-Bottled water |
| | | | | | 8-Water vendor |
| | | | | | 9-Stored in reservoir |
| | | | | | 10-Pond/canal/river |
| | | | | | 11-Shared tab/tube well/well in HH |
| | | | | | 77-Others |
| Datafile | TypeDW | Type of drinking water | Numeric | 1 | 1-Boiled |
| | | | | | 2-Filtered |

| Table | Variable name | Description of variable | Data type | Width | Coding category of variable |
|---|---|---|---|---|---|
| | | | | | 3-Chemicals treated<br>4-Not treated<br>9-Do not know |
| Datafile | Totalc | Current value/monthly rent | Numeric | 7 | Exact value<br>9999999-Do not know |
| Datafile | WaFilt | Is there a water filter in the HH? | Numeric | 1 | 1-Yes<br>2-No |
| Datafile | FiltWa | Is there any water in the filter device? | Numeric | 1 | 1-Yes<br>2-No<br>3-Refused |
| Datafile | WaChem | Is there any water treatment chemical in the HH? | Numeric | 1 | 1-Yes<br>2-No<br>3-Refused |
| Datafile | HandWa | Is hand washing water available at the visiting time? | Numeric | 1 | 1-Yes<br>2-No<br>3-Refused |
| Datafile | Soap | Is hand washing soap available at the visiting time? | Numeric | 1 | 1-Yes<br>2-No<br>3-Refused |
| Datafile | Pop100m | Population within 100m around the household | Numeric | 6 | Exact value |
| Datafile | Schp100m | Population 12 years and older within 100m around the household | Numeric | 6 | Exact value |
| Datafile | Edu100m | # individuals with at least 5 years of schooling within 100m around the household | Numeric | 6 | Exact value |
| Datafile | Dw100m | # of households having safe water drinking source within 100m around the household | Numeric | 6 | Exact value |
| Datafile | Wat100m | # of household using treated water within 100m around the household | Numeric | 6 | Exact value |
| Datafile | Chp100m | # of children under five within 100m around the household | Numeric | 6 | Exact value |

| | | | | | |
|---|---|---|---|---|---|
| Datafile | Cad100m | # of children using sanitary toilet within 100m around the household | Numeric | 6 | Exact value |
| Datafile | Adp100m | # of individuals 5 years or older within 100m around the household | Numeric | 6 | Exact value |
| Datafile | Tad100m | # of individuals 5 years or older using sanitary toilet within 100m around the household | Numeric | 6 | Exact value |
| Datafile | Hospdist | Distance from household to the nearest treatment center (meter) | Numeric | 6 | Exact value |
